# Its Coat of Arms and Badge. An Attractive Competition

**Published:** 1919-08-16

**Authors:** S. D. Clippingdale


### THE EDITOR'S LETTER-BOX.

[Correspondence on all subjects is invited, but we cannot in any way be responsible for the opinions expressed by our correspondents, who must give their name and address as a guarantee of good faith, but not necessarily for publication. Correspondents are reminded that brevity of style and conciseness of statement greatly facilitate early insertion.]

### THE COLLEGE OF NURSING.

### Its Coat of Arms and Badge. An Attractive Competition.

*To the Editor of* The Hospital.

Sir,—I see the College of Nursing is advertising to the *public* for a Coat of Arms. It seems to me incredible that the College cannot know that the proper way to obtain a grant is by aplication to Herald's College. Anything else is bogus, void, and of no effect. Of course the College of Arms would charge a fee (about £80) but it seems to me it would be better to pay this than to distribute money among amateurs. The College of Arms is always ready to consult a grantee with regard to the "Bearings" upon a shield. In *The Antiquary* for November and December, 1915, I published under the title "Heraldry and Medicine" what I believe is the only paper written in this country upon the application of Heraldry to Medicine, and I should be very glad to give the Council of the College of Nursing any help I could in the matter. At all events, I would strongly and respectfully urge the Council, while they are about it to get a *genuine* coat, a coat which would serve for all time and from which a badge and "livery" colours could be legally devised.—Yours faithfully,

S. D. Clippingdale.

### The Interest Spreading throughout the Empire.

* * * Dr. S. D. Clippingdale's letter demonstrates that he could not have read with understanding the announcement of The College of Nursing on page 482 of The Hospital of the 9th inst. This notice relates to its Coat of Arms and Badge, announces a competition and opens with the words "It is proposed to apply for a Coat of Arms and Badge." That, of course, means that preliminary steps with this in view, on the lines indicated by Dr. Clippingdale, have been taken "to get a genuine Coat, a Coat which would serve for all time and from which a badge and colours could be legally devised."

Meanwhile there are at least 50,000 fully-qualified nursing sisters and matrons who take a keen interest in the College, its Coat of Arms and Badge, and everything connected with it. The same may be said of a large number of members of the public in various ranks of life, including the leisured and intellectual classes and artists. The Coat of Arms and Badge will be something that will be honoured by the people throughout the Empire, and the College has undoubtedly taken the right course, as the first step in this important development, to announce an attractive competition with the hope, and we believe the certainty, that a number of highly-qualified and capable competitors will gladly take part in the competition. Thus the outcome ought to be of permanent value and international interest. We hope that before September 30 next, by which date the designs and suggestions must be received, this forecast of the probable results of this attractive competition will be abundantly fulfilled. If Dr. S. D. Clippingdale would send us *The Antiquary* for November and December-1915, containing his article on "Heraldry and Medicine" we shall welcome it, take care of his copies, and return them in due course. He may thus extend the interest in the competition and do the College of Nursing and Trained Nurses, generally, a substantial service.—Ed. The Hospital.

